# Altered long non-coding RNAs predict worse outcome in osteosarcoma patients: evidence from a meta-analysis

**DOI:** 10.18632/oncotarget.16470

**Published:** 2017-03-22

**Authors:** Yanliang Yang, Shunli Wang, Teng Li

**Affiliations:** ^1^ Bone Tumour & Bone Disease Department I, Zhengzhou Orthopaedics Hospital, Zhengzhou Orthopedics Hospital Affiliated to Henan University, Henan, China

**Keywords:** osteosarcoma, lncRNA, prognosis, meta-analysis

## Abstract

Long non-coding RNAs (lncRNAs) are emerging as promising prognostic biomarkers in an expanding list of malignant neoplasms. Here, we sought to investigate the strength of associations between lncRNA signatures and clinical outcomes in osteosarcoma. We conducted a systematic search of the online databases from inception to July 2016. Hazard ratios (HRs) with corresponding 95% confidence intervals (CIs) for the primary endpoints of overall survival (OS), progression-free survival (PFS) or event-free survival (EFS) were extracted and meta-analyzed. Our results manifested that altered lncRNAs expression was markedly associated with worse OS (univariate analysis: HR = 3.20, 95% CI: 2.42-4.24, *P* = 0.000; multivariate analysis: HR = 2.66, 95% CI: 1.92-3.69, *P* = 0.000), PFS (HR = 2.05, 95% CI: 1.32-3.18, *P* = 0.001) and EFS (HR = 4.37, 95% CI: 1.64-11.66, *P* = 0.003) times among osteosarcoma patients. In the pooled analyses stratified by clinicopathological features, levels of lncRNAs were closely correlated with tumor size (pooled *P* = 0.001), tumor stage (pooled *P* = 0.003), and distant metastasis (pooled *P* = 0.002) in osteosarcoma. The results obtained in our work suggest that altered lncRNA signatures predict unfavorable clinical outcomes and are acceptable to be potential prognostic biomarkers in forecasting prognosis of osteosarcoma.

## INTRODUCTION

Osteosarcoma represents 55% of all specified malignant bone cancers in adolescences under the age of twenty [1]. More than 20% of the young-onset osteosarcoma patients present with distant metastases at diagnosis, and 40% cases in advanced stages progress to metastasis during therapy [2]. Despite the developments of novel treatment strategies, patients suffered from osteosarcoma still evolve with a dissatisfying prognosis. It is reported that the 5-year survival rate of osteosarcoma is lower than 62% in patients with localized disease, yet in those with recurrent or metastatic status, this rate will be attenuated to about 20% [1,3]. In consequence, there is a critical need to find and develop novel prognostic biomarkers in monitoring progression and survival of osteosarcoma in clinic.

Recently, the discovery of the long non-coding RNAs (lncRNAs) has provided new insights into cancer research. LncRNA is defined as one kind of endogenous RNA comprises a sequence larger than 200 nucleotides but with no significant or functional open reading frame(s) [4]. Since their discoveries, lncRNAs are being investigated at a rapid pace and the corresponding functions are being interpreted. At present, lncRNAs have been confirmed to be implicated in regulation of diverse biological processes, including gene expression, cell proliferation, apoptosis, migration, and protein localization, etc [5–7]. More than that, many investigations have indicated promising results for the potential value of lncRNAs as prognostic indicators in cancers [8–11]. For instance, a meta-analysis from Chen et al reported that altered lncRNAs were not only associated with poor prognoses in renal cell carcinoma cancer, but also correlated to lymph node metastasis and distant metastasis of the disease, thereby recommending their potential clinical applications as novel biomarkers in forecasting prognosis or guiding therapeutics [11].

Till now, the prognostic roles of multi-types of lncRNAs have been investigated in osteosarcoma as well, consisting of HULC, HOTTIP, MEG3, TUSC7, TUG1, 91H, and OMRUL, etc [12–23]. All of the above-mentioned lncRNA signatures have in common that testing of lncRNA(s) levels may be served as potential and efficient prognostic markers for osteosarcoma. Nevertheless, no meta-analysis has been conducted to provide a comprehensive overview of the clinical utilities of lncRNAs in surveillance of osteosarcoma prognosis. Herein, we established standardized inclusion criteria and statistical approach, and conducted this meta-analysis with the purpose of giving a comprehensive evaluation of the associations between lncRNA signatures and clinical outcomes in osteosarcoma.

## RESULTS

### Study selection and characteristics

**Figure 1:**
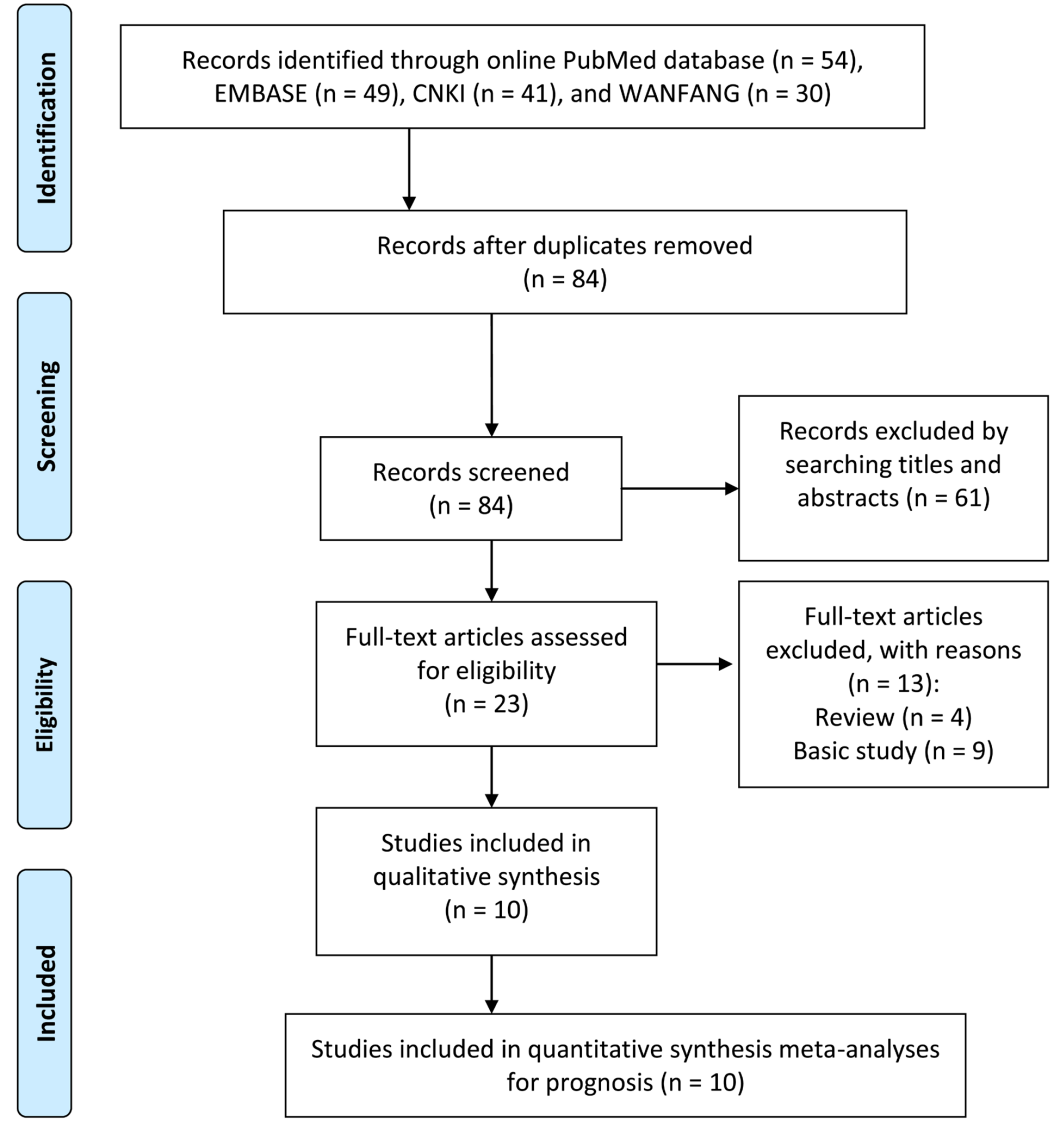
Flow diagram of study selection procedure

The procedure of literature inclusion and exclusion is illustrated in Figure 1. According to the predefined criteria, a total of 84 records were obtained from the online PubMed, EMBASE, Chinese National Knowledge Infrastructure (CNKI), and WANFANG databases after duplicates removed. After the manual screening, 61 citations were excluded by searching titles and abstracts. Twenty three articles received full-text evaluation, and 13 publications were discarded either because the status of reviews or basic research articles. Finally, a total of 10 cohorts were evaluated for the final meta-analysis.

The major characteristics of each enrolled study are summarized in Table 1. Ten publications with a combined study population of 674 cases were included. All samples were retrieved prior to treatment and the final diagnosis all relied on the histopathological examinations. Expression status of all lncRNAs was determined by quantitative reverse transcription polymerase chain reaction (qRT-PCR). The reference genes comprised DAPGH [14, 17, 19–21, 23], G3PDH [22], β-actin [13, 16, 21], and 18S rRNA [18]. The primary endpoints included OS, PFS, and EFS, with a median follow-up time ranged from 25 to 60 months. The altered lncRNA expression profile possessed a total of 9 individuals, yet 7 of them (TUG1, 91H, HULC, BANCR, FGFR3-AS1, HOTTIP, and OMRUL) were overexpressed in osteosarcoma tissues/plasma, and 2 (MEG3 and TUSC7) were down-regulated.

**Table 1:** Summary of lncRNAs used as prognostic biomarkers in forecasting osteosarcoma

Author	Year	Area	Study design	Patient size	Tumor stage I+II (%)	LncRNAs		Sample type	Test method	Reference gene	Survival indicator	HR&95%CI availability	Follow-up months	NOS score
						Signature	Expression							
Cong et al [13]	2016	China	R	82	39.0	TUSC7	Decreased	Tissue	qRT-PCR	β-actin	OS	Directly	Unclear	7
Li et al [14]	2015	China	R	68	44.1	HOTTIP	Increased	Tissue	qRT-PCR	GAPDH	OS	Directly	60	7
Ma et al. [16]	2016	China	R	76	84.2	TUG1	Increased	Tissue	qRT-PCR	β-actin	OS, PFS	Directly	Median: 44, 24	8
Peng et al [17]	2016	China	R	84	68.4	BANCR	Increased	Tissue	qRT-PCR	GAPDH	OS	Indirectly	Unclear	7
Sun et al [18]	2016	China	R	62	65.6	FGFR3-AS1	Increased	Tissue	qRT-PCR	18S rRNA	OS	Indirectly	Median: 31	8
Sun et al [19]	2015	China	R	78	44.9	HULC	Increased	Tissue	qRT-PCR	GAPDH	OS	Directly	Unclear	7
Tian et al [20]	2015	China	R	64	48.4	MEG3	Decreased	Tissue	qRT-PCR	GAPDH	OS	Directly	10-60	7
Uzan et al [21]	2016	Brazil	R	33	70.0	HULC	Increased	Tissue	qRT-PCR	GAPDH & actin	OS, EFS	Indirectly	96	8
Xia et al [22]	2016	China	R	67	83.6	91H	Increased	Plasma	qRT-PCR	G3PDH	OS	Directly	60 (3-60)	8
Zhu et al [23]	2015	China	R	60	Unclear	OMRUL	Increased	Tissue	Microarray	GAPDH	OS	Indirectly	25 (6-96)	7

R: retrospective study;OS: overall survival; PFS: progression-free survival; EFS: event-free survival.

### Article quality and heterogeneity

Article quality was judged by the NOS checklist [24], and total evaluation scores of each study regarding cohort selection, comparability and outcome ascertainment are summarized in Table 1. All studies retained NOS scores larger or equal to 7, suggesting a relatively high quality of the included investigations.

For the heterogeneity analysis checked by I-squared and Chi-squared tests, we only observed a slight degree of heterogeneity in the analysis of pooled EFS (I-squared = 57.8%), whereas other combined analyses involveded pooled of OS and PFS times, all showed no significant heterogeneity existed among studies. Additionally, heterogeneity appeared in the stratified analyses as Chemotherapy-based (I-squared = 90.1%, *P* = 0.001 in Chi-squared test) and down-regulated lncRNAs (I-squared = 83.2%, *P* = 0.003 in Chi-squared test) analyses (Table 2), and therefore the DerSimonian and Laird method was employed for statistical analysis.

**Table 2:** Associations observed between clinicopathological variables and OS time in osteosarcoma

Factors	Univariate analysis				Multivariate analysis			
	Pooled HR (95%CI)	*P* value	Heterogeneity		Pooled HR (95%CI)	*P* value	Heterogeneity	
			I-squared (%)	Chi-squared (*P*)			I-squared (%)	Chi-squared (*P*)
Clinicopathological features								
Age	1.11 (0.86-1.45)	0.420	0.0	0.773				
Gender	1.06 (0.83-1.37)	0.623	0.0	0.879				
Anatomic location	1.00 (0.76-1.32)	0.996	0.0	0.783				
Tumor size	2.03 (1.43-2.88)	0.000	0.0	0.773				
Tumor stage	2.86 (2.17-3.77)	0.000	0.0	0.740	2.69 (2.01-3.59)	0.000	19.1	0.284
Distant metastasis	3.64 (2.70-4.91)	0.000	0.0	0.571	3.35 (2.48-4.51)	0.000	0.0	0.780
Chemotherapy	1.02 (0.18-5.60)*	0.987*	90.1	0.001	1.28 (0.72-2.27)	0.396	54.0	0.140
Expression status								
Up-regulated lncRNAs	3.24 (2.38-4.40)	0.000	0.0	0.813	2.17 (1.90-3.87)	0.000	0.0	0.979
Down-regulated lncRNAs	1.52 (0.35-6.61)*	0.579*	83.2	0.003				

HR: hazard ratios; CI: confidence intervals. *indicates that data were obtained by using a random-effect model in the meta-analysis.

### Results of the prognostic meta-analysis

**Figure 2:**
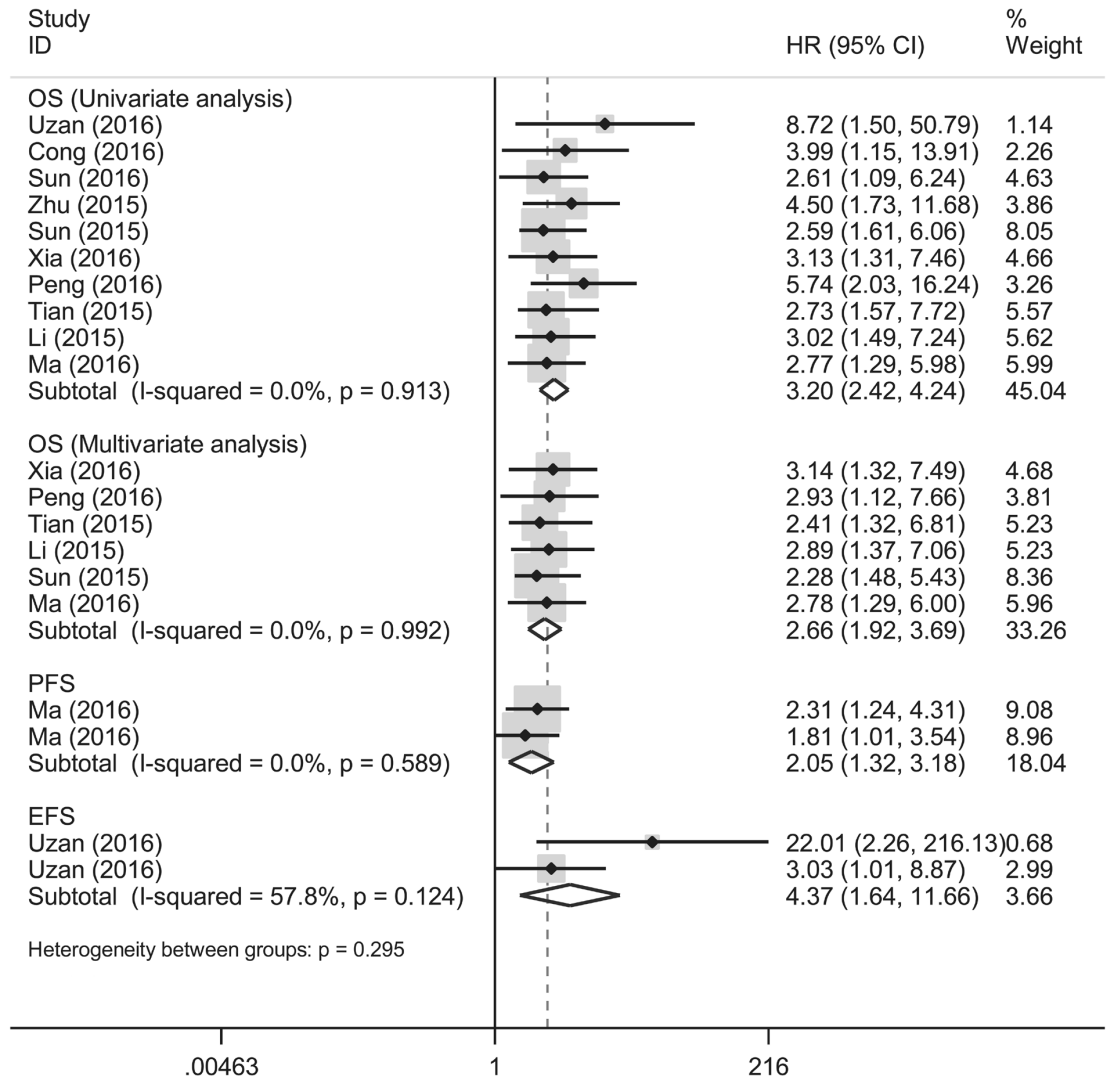
Prognostic utilities of lncRNA signatures in predicting OS, PFS and EFS times in osteosarcoma

Our results manifested that altered lncRNA profiles were significantly associated with poor OS (univariate analysis: HR = 3.20, 95% CI: 2.42-4.24, *P* = 0.000; multivariate analysis: HR = 2.66, 95% CI: 1.92-3.69, *P* = 0.000), and also influenced both PFS (HR = 2.05, 95% CI 1.32-3.18, *P* = 0.001) and EFS (HR = 4.37, 95% CI: 1.64-11.66, *P* = 0.003) among osteosarcoma patients (Figure 2).

### Subgroup analysis

In the subgroup analysis stratified by clinicopathological features, significant negative associations were observed between OS time and the following variables: tumor size (univariate analysis: HR = 2.03, 95% CI: 1.43-2.88, *P* = 0.000), clinical stage (univariate analysis: HR = 2.86, 95% CI: 2.17-3.77, *P* = 0.000; multivariate analysis: HR = 2.69, 95% CI: 2.01-3.59, *P* = 0.000), and distant metastasis (univariate analysis: HR = 3.64, 95% CI: 2.70-4.91, *P* = 0.000; multivariate: HR = 3.35, 95% CI: 2.48-4.51, *P* = 0.000) (Supplementary Figure 1 to 3 and Table 2). Moreover, for the analyses based on expression status, the up-regulated lncRNAs were more likely to manifest shorter OS time (univariate analysis: HR = 3.24, 95% CI: 2.38-4.40, *P* = 0.000; multivariate analysis: HR = 2.17, 95% CI: 1.90-3.87, *P* = 0.000) compared with the down-regulated lncRNAs in osteosarcoma (Supplementary Figure 1 to 3 and Table 2). Differences across other clinicopathological variables, including age, gender, anatomic location, chemotherapy and decreased lncRNA expression were not significant.

Comparison of the *P* values of correlations between lncRNA signatures and clinicopathological features are documented in Table 3 and Supplementary Table 1. The data showed that lncRNAs expression was significantly correlated with tumor size (Chi-squared = 39.12, pooled *P* = 0.001), tumor stage (Chi-squared = 65.14, pooled *P* = 0.003), and distant metastasis (Chi-squared = 65.93, pooled *P* = 0.002) in osteosarcoma, but was not significantly linked to gender, age, and anatomic location (Supplementary Table 1)

**Table 3:** Comparison of the P values of correlations between lncRNA signature and clinicopathological features in osteosarcoma

Study	Area	LncRNAs		Patient size	Gender	Age	Tumor size (cm)	Tumor stage	Anatomic location	Distant metastasis	Chemotherapy (Yes or No)
		Signature	OS								
Cong 2016 [13]	China	TUSC7	0.030	82	0.650	0.473	NM	0.294	0.627	0.087	NM
Li 2015 [14]	China	HOTTIP	0.007	68	0.465	0.215	0.120	0.003	0.161	0.016	NM
Ma 2016 [16]	China	TUG1	0.009	76	0.885	0.318	0.044	0.082	0.769	0.015	0.012
Peng 2016 [17]	China	BANCR	0.028	84	0.509	0.505	0.008	0.004	0.814	0.02	NM
Sun 2016 [18]	China	FGFR3-AS1	0.031	62	0.611	0.309	0.041	0.013	0.490	0.030	NM
Sun 2015 [19]	China	HULC	0.009	78	0.492	0.352	0.496	0.003	0.624	0.005	NM
Tian 2015 [20]	China	MEG3	0.006	64	0.614	0.302	0.076	0.006	0.281	0.011	NM
Uzan 2016 [21]	Brazil	HULC	0.016	33	0.999	0.065	0.670	0.999	0.274	0.999	NM
Xia 2016 [22]	China	91H	0.01	67	0.806	0.738	0.073	0.106	0.653	0.007	0.030
Zhu 2015 [23]	China	OMRUL	0.002	60	NA	0.100	NM	NM	0.070	NM	NM

NM: not mentioned

### Influence analysis and publication bias

**Figure 3:**
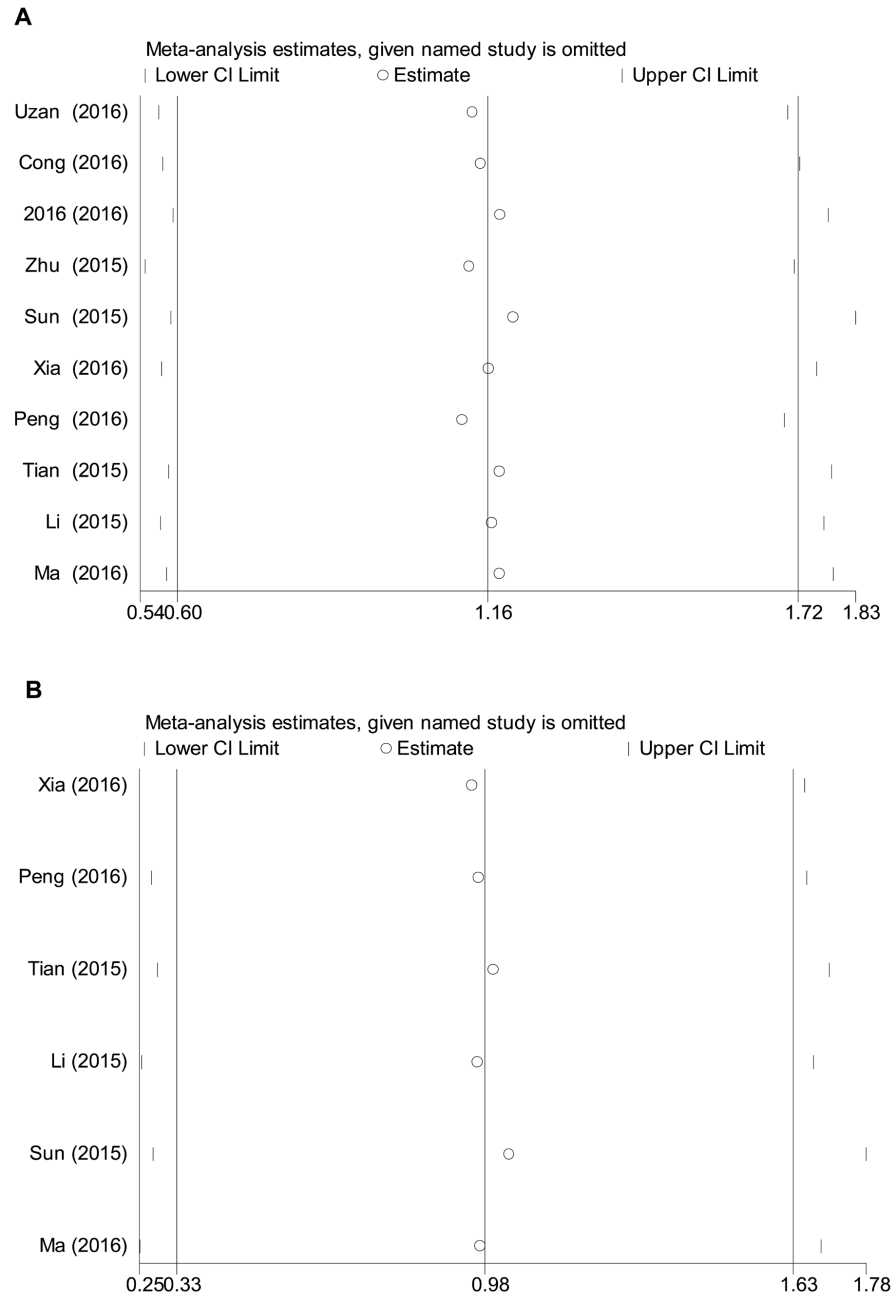
Influence analysis of the pooled studies **A**. univariate analysis of the pooled HRs for OS time; **B**. multivariate analysis of the pooled HRs for OS time.

Sensitivity analysis were undertaken to deeply trace the outliers among studies. As indicated in Figure 3, no individual studies were assessed as outliers, hinting that the pooled results of our meta-analyzed data were reliable.

**Figure 4:**
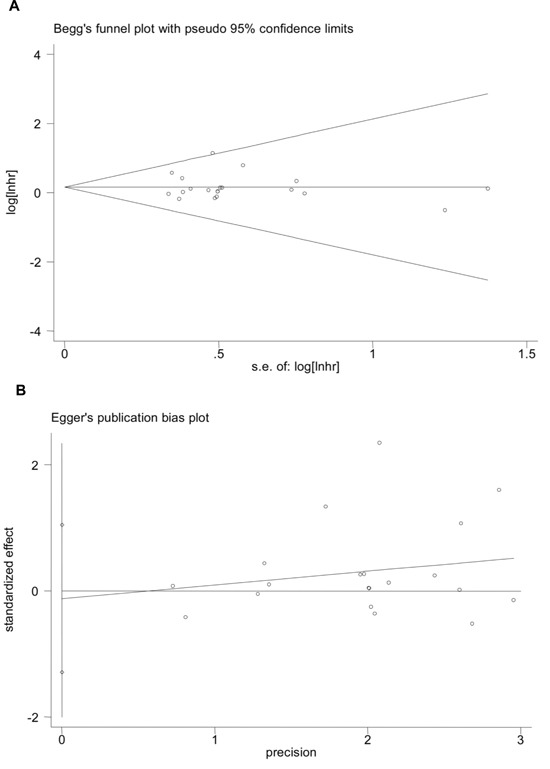
Publication bias of the overall pooled analyses evaluated by Bgger's funnel plot and Egger's publication bias plot **A**. Bgger's funnel plot (*P* = 0.442); **B**. Egger's publication bias plot (*P* = 0.142).

Degrees of publication bias were judged by Bgger's funnel plot and Egger's publication bias plot. As exemplified in Figure 4, both the two tests presented *P* values larger than 0.5 in the overall pooled analyses, suggesting a low likelihood of significant bias due to article publication.

## DISCUSSION

Osteosarcoma is a complex and aggressive primary bone sarcoma, making up 55% of the specified bone cancers in adolescents with 0 to 19 years of age [1]. Despite the rapid advantages in diagnosis and therapeutics, the prognostic outcomes of osteosarcoma remain dissatisfying [1, 3]. It is urgent to identify and develop novel markers or targets to aid in diagnosis, treatment, as well as prognosis of osteosarcoma. In recent years, lncRNAs have been highlighted as potential prognostic biomarkers for osteosarcoma [12–23]. In this respect, a comprehensive meta-analysis is warranted to present an overview of the prognostic utilities of lncRNAs for osteosarcoma. We therefore conducted this meta-analysis, attempting to assess the associations between lncRNA signatures and survival times in osteosarcoma.

Our results demonstrated that altered lncRNA signatures were significantly associated with shorter OS, PFS as well as EFS times in osteosarcoma. The pooled HR of OS time for univariate analysis was estimated to be 3.20 (*P* = 0.000), and that for multivariate analysis was 2.66 (*P* = 0.000), hinting that altered lncRNA expression, at least the current well-characterized lncRNAs, are acceptable to be novel indicators in forecasting prognosis of osteosarcoma. Similar results were obtained in merged PFS and EFS of the osteosarcoma patients as well. A newly published meta-analysis study from Chen et al evidenced that lncRNAs could act as promising markers for prognosis as well as lymph node metastasis and distant metastasis in renal cell carcinoma [11]. Another pooled analysis conducted by Hong et al also manifested that lncRNA-UCA1 expression status is markedly correlated with poor OS and PFS times in various cancers [25]. Agree with the above findings, our data strengthened the observation that lncRNA profiles sustain a pivotal role in forecasting prognosis in osteosarcoma.

LncRNAs are involved in the carcinogenesis as well as progress and metastasis of osteosarcoma [14, 16, 17, 19, 20, 22]. In our subgroup analysis stratified by clinicopathological features, we found that tumor size, clinical stage and distant metastasis were negatively associated with OS time of the osteosarcoma patients. More importantly, lncRNA profiling was found to be strongly correlated with tumor size, clinical stage and distant metastasis. The results allowed us to suggest that lncRNA(s) expression is correlated to osteosarcoma with a more aggressive behavior. A study meta-analyzed the associations between lncRNA-MALAT1 expression and clinicopatholoical characteristics corroborates our findings: MALAT1 level was demonstrated to be closely associated with clinical stage and lymph node metastasis in renal cell carcinoma [11]. Another investigation from Jing et al presented the similar results [26]. Nevertheless, some research found no significant associations between lncRNA-HULC expression and clinicopathology features in osteosarcoma [21]. Thus, more evidences are still warranted to reinforce our findings.

Among the altered lncRNA profiles, 7 lncRNAs involved TUG1, 91H, HULC, BANCR, FGFR3-AS1, HOTTIP and OMRUL [14, 16–19, 21–23], were notably increased in patients with osteosarcoma and strongly correlated to worse clinical outcomes, indicating that such lncRNAs may play oncogenic roles in maintaining tumor progression. Intriguingly, Zhang's investigation had evidenced that some of the above lncRNAs, such as TUG1, was down-regulated in non-small cell lung carcinoma and also indicated poor survival in such disease [27]. On the other hand, abundance of two types of lncRNAs, MEG3 and TUSC7 were demonstrated to be down-regulated in osteosarcoma patients and significantly associated with survival times [13, 20]. It has been reported that expression of MEG3 RNA was detectable in many normal tissues, but lost or decreased in many primary cancers [28]. Similarly, expression level of TUSC7 was lower in osteosarcoma tissues than non-tumor tissues, and that silencing of TUSC7 expression in osteosarcoma cells resulted in promoted cell viability [13]. These findings indicate that expression of lncRNAs may be tissue-specific and act distinct but different roles in cancer biology.

Despite the promising data, the current study still yields some limitations. Firstly, a total of 9 individual lncRNAs were included and the pooled results, to some extent, only provide an overview of the prognostic utilities of all current studied lncRNAs, yet suitable lncRNA(s) or expression patterns or other novel lncRNAs for clinical applications should be further identified and confirmed. Secondly, the enrolled studies encountered restricted patient sizes, which, to some extent, may compromise the overall accuracy of the pooled results. Thirdly, most of studies were conducted in Chinese and the results may be more applicable to this racial population group. Last, many factors included different cut-off points for lncRNA signatures may eventually increase the heterogeneity among studies; additionally, different clinical stages, treatments (surgical remission), chemotherapy responses, as well as unclear concomitant disease conditions among patients, may eventually affect the endpoints of survival in osteosarcoma.

Taken together, the current study demonstrated that lncRNA signatures hallmark promising value in surveillance of clinical outcomes, and therefore could be developed as predictive biomarkers for osteosarcoma survival. Notwithstanding, other large-scale and high quality investigations are still warranted to further validate the clinical utilities of lncRNAs (single or in parallel) for osteosarcoma.

## MATERIALS AND METHODS

### Publication search

The entire contents of this study are in compliance with the guidelines of Preferred Reporting Items for Systematic Reviews and Meta-analysis (PRISMA) [29]. We conducted a computerized literature search in the online PubMed, EMBASE, CNKI, and WANFANG databases from inception to July 31^st^ 2016. The search keywords were predefined as “long non coding RNA” or “lncRNA”, “osteosarcoma” or “osteogenic sarcoma”, and “prognosis” or “hazard ratio” or “HR”, etc. To avoid missing eligible studies, we manually searched the article references for data collection as well.

### Eligibility criteria

The inclusion criteria were: (a) cohort design; (b) studies addressed the association between lncRNA(s) expression and prognosis of osteosarcoma; (c) the primary endpoints as OS, PFS, DFS (disease-free survival) or EFS were clearly defined; and (d) the HRs with corresponding 95%CIs were available or can be calculated indirectly. The exclusion criteria were: (a) studies unrelated to the prognostic utility of lncRNA(s) in osteosarcoma; (b) data was unclearly presented and insufficient to perform the statistical analysis, and (c) basic research, animal studies, reviews, letters and comments, etc.

### Data extraction and quality assessment

Two authors (S. Wang and T. Li) independently extracted the data with the contents of name of the first author, country, publication date, study design, ethnicity, patient size, sample type, lncRNA profile, test method, survival endpoints (OS, PFS, DFS or EFS), follow-up months, HRs and 95%CIs, and *P* values, etc. Differences in opinion from the reviewers were finally resolved by group consensus.

Study quality of all cohort studies was judged utilizing the Newcastle-Ottawa Scale (NOS) checklists, wherein, cohort selection, comparability and outcome ascertainment of each study were estimated, with a maximum evaluation score of 9 (a score ≥ 6 was rated as study with high quality)[24]. If the enrolled single study received a relatively low evaluation score after quality assessment, it will be excluded for the final statistical analysis.

### Statistical analysis

All statistical analyses were undertaken via Stata 12.0 program (Stata Corporation, College Station, TX, USA). Degrees of study heterogeneity was examined by Chi-squared and I-squared tests, and significant level was set at *P*<0.05 (I-squared>50%) [30]. The DerSimonian and Laird method (random-effect model) will be applied in case of significant heterogeneity existed among studies, otherwise, the Mantel-Haenszel method (fixed-effect model) will be chosen for the analysis [31]. For the pooled analysis, HRs and the corresponding 95% CIs were extracted and meta-analyzed for aggregation of the survival results. In the stratified analysis, the *P* values were merged by utilizing Fisher's test. Publication bias was checked by Egger's and Bgger's tests, and *P*<0.05 was deemed as statistical significant [32].

## SUPPLEMENTARY MATERIALS FIGURES AND TABLES


