# Supplementary material for: Altered long non-coding RNAs predict worse outcome in osteosarcoma patients: evidence from a meta-analysis
Source: Oncotarget. 2017 Mar 22;8(21):35234–43. doi: 10.18632/oncotarget.16470 (PMC5471049; doi:10.18632/oncotarget.16470)
Supplement: Supplementary file 1 [file oncotarget-08-35234-s001.pdf]

Altered long non-coding RNAs predict worse outcome in osteosarcoma patients: evidence from a meta-analysis

Supplementary Material

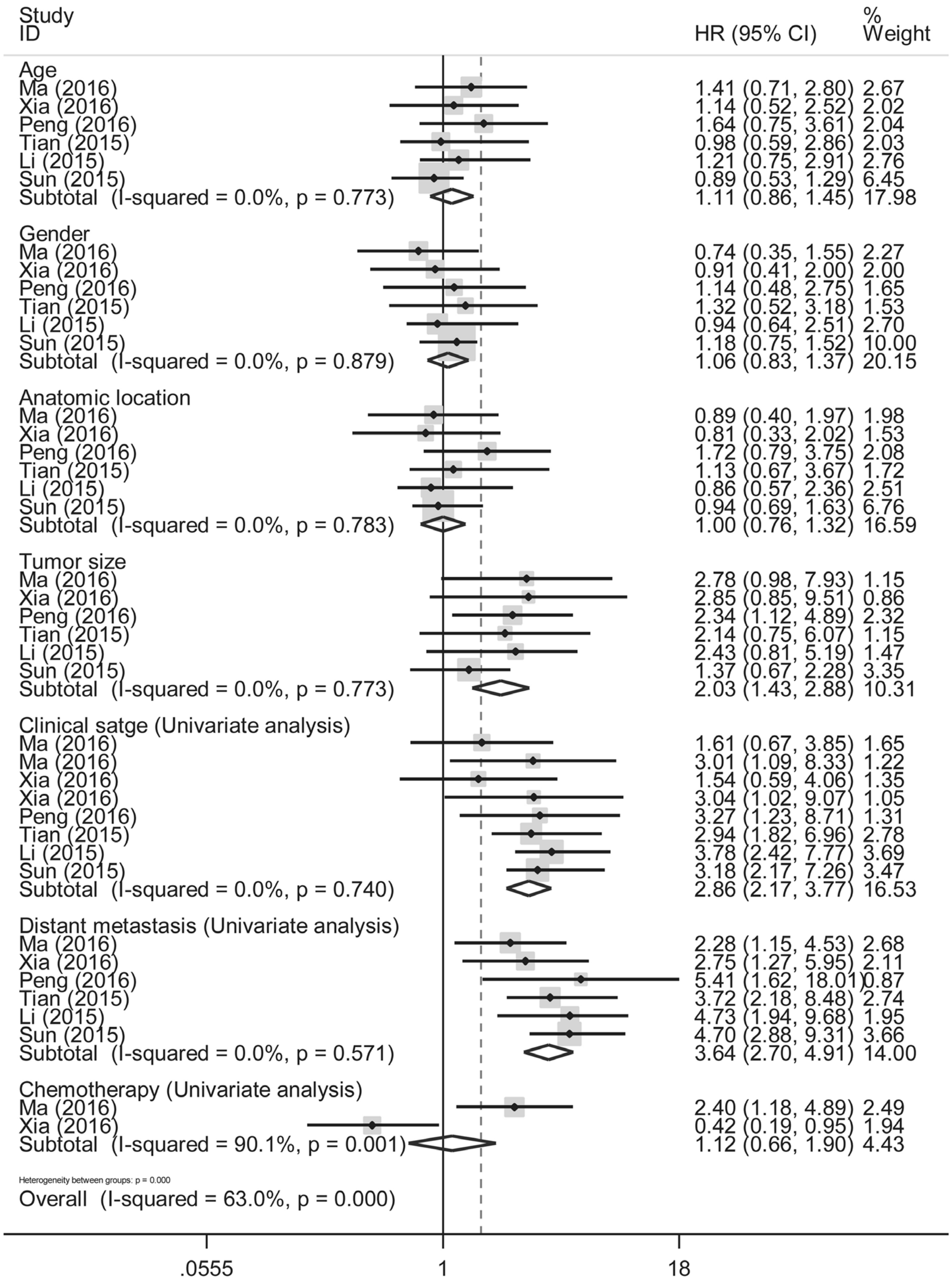

Supplementary Figure 1: Univariate analysis of the clinicopathological factors in predicting OS time in osteosarcoma.

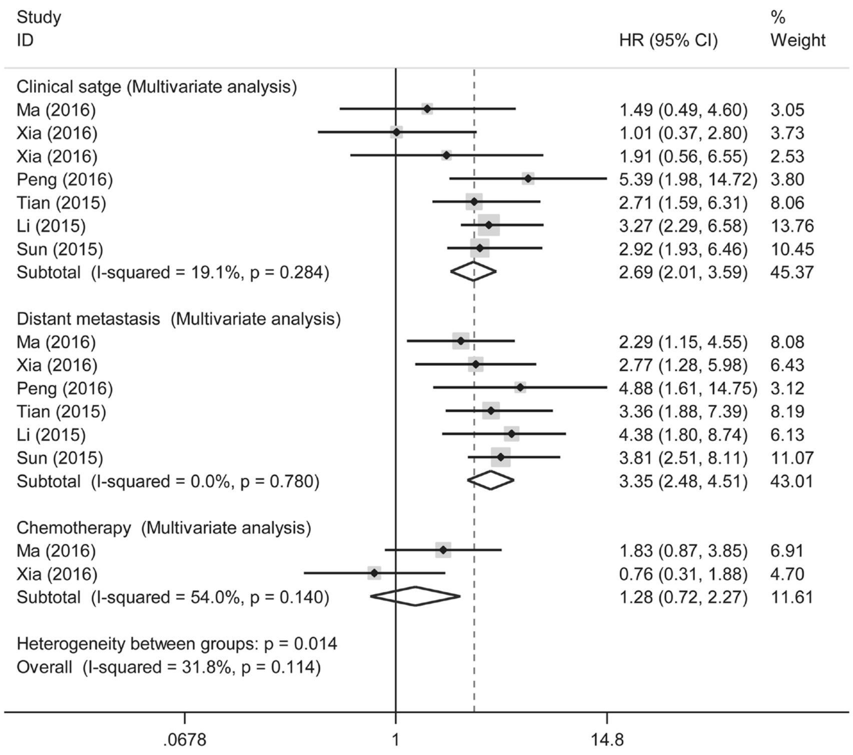

Supplementary Figure 2: Multivariate analysis of the clinicopathological factors in predicting OS time in osteosarcoma.

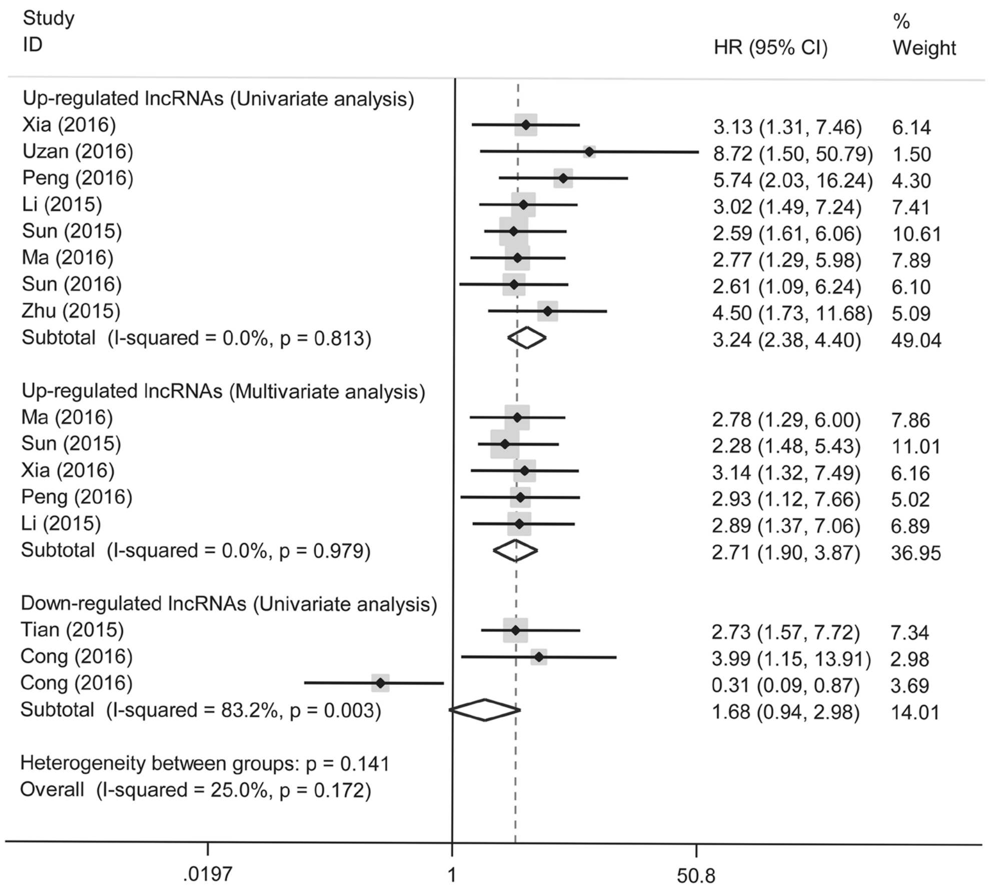

Supplementary Figure 3: Associations between lncRNA expression status and OS time in osteosarcoma.

Supplementary Table 1: The pooled P values of correlations between lncRNAs expression and clinico-pathological features in osteosarcoma

Factors	Included studies	Included lncRNAs	Method	Chi-squared value	Pooled *P* value
Gender	9	8	Fisher's test	7.80	0.982
Age	10	9	Fisher's test	25.74	0.173
Tumor size	8	7	Fisher's test	39.12	0.001
Anatomic location	10	9	Fisher's test	19.19	0.509
Enneking stage	9	8	Fisher's test	65.14	0.003
Distant metastasis	9	8	Fisher's test	65.93	0.002
